# Supplementary material for: Seroprevalence of West Nile Virus among Equids in Bulgaria in 2022 and Assessment of Some Risk Factors
Source: Vet Sci. 2024 May 9;11(5):209. doi: 10.3390/vetsci11050209 (PMC11126025; doi:10.3390/vetsci11050209)
Supplement: Supplementary file 1 [file vetsci-11-00209-s001.zip › vetsci-2947733-supplementary.pdf]

Table S1. Distribution of the tested samples by province, municipality, region, species and sex.

Province	Municipality	Region	N				
			samples	horses	donkeys	males	females
Stara Zagora	Stara Zagora		15	9	6	5	10
	Kazanlak		14	13	1	9	5
	Maglizh		5	5	–	2	3
Total	3	SE	34	27	7	16	18
Burgas	Burgas		4	4	–	3	1
	Ruen		27	27	–	–	27
	Tsarevo		9	9	–	6	3
Total	3	SE	40	40	–	9	31
Plovdiv	Plovdiv		1	1	–	–	1
Total	1	SC	1	1	–	–	1
Haskovo	Haskovo		2	2	–	2	–
	Svilengrad		64	64	–	6	58
Total	2	SC	66	66	–	8	58
Smolyan	Dospat		5	5	–	3	2
	Devin		11	11	–	5	6
	Chepelare		2	2	–	2	–
Total	3	SC	18	18	–	10	8
Blagoevgrad	Satovcha		18	8	10	13	5
	Hadzhidimovo		21	9	12	15	6
Total	2	SW	39	17	22	28	11
Pernik	Pernik		5	5	–	2	3
Total	1	SW	5	5	–	2	3
Sofia city	Kremikovtsi		26	4	22	–	26
Total		SW	26	4	22	–	26
Sofia province	Ihtiman		18	18	–	7	11
	Samokov		13	–	13	7	6
	Koprivshtitsa		43	43	–	13	30
Total	3	SW	74	61	13	27	47

Montana	Valchedram		39	39	–	16	23
Total	1	NW	39	39	–	16	23
Vratsa	Mizia		11	11	–	3	8
Total	1	NW	11	11	–	3	8
Pleven	Nikopol		1	1	–	1	–
Total	1	NC	1	1	–	1	–
Gabrovo	Gabrovo		3	3	–	2	1
Total	1	NC	3	3	–	2	1
Dobrich	Dobrich		11	11	–	7	4
Total	1	NE	11	11	–	7	4
Varna	Varna		10	10	–	6	4
Total	1	NE	10	10	–	6	4
**Total**	**25**	**6**	**378**	**314**	**64**	**135**	**243**
